# Development of a revised version of the transdiagnostic skills scale (T2S-R)

**DOI:** 10.3389/fpsyg.2024.1290692

**Published:** 2024-02-12

**Authors:** Alexis Vancappel, Nicolas Gillet, Evelyne Fouquereau, Séverine Chevalier, Julia Aubouin-Bonnaventure, Hélène Coillot, Anna Mangolini, Eline Jansen, Cinzia Dicosimo, Wissam El-Hage

**Affiliations:** ^1^Département de Psychologie, QualiPsy, Qualité de Vie et santé Psychologique, Université de Tours, Tours, France; ^2^CHRU de Tours, Pôle de Psychiatrie-Addictologie, Tours, France; ^3^Institut Universitaire de France, Paris, France; ^4^UMR 1253, iBrain, Université de Tours, Inserm, Tours, France

**Keywords:** transdiagnostic, CBT, psychological skill, psychometric, psychiatric disorder

## Abstract

**Introduction:**

The transdiagnostic approach has been shown to offer promising prospects in psychopathology, based on the observation that common factors may be involved in different psychiatric disorders. The transdiagnostic skills scale (T2S) was developed recently to assess the skills that are disrupted in these disorders. However, studies have shown that the T2S has lower predictive power for externalizing than internalizing disorders. This may be due to the fact that the skills assessed do not include the control of urges and cravings. The aims of the current study are thus to develop a revised version of the T2S (T2S-R) integrating this dimension, and to assess its factor structure and invariance across employment status (workers vs. students) and the level of psychopathology.

**Method:**

We recruited 1,298 French participants online through social media. They completed the revised version of the T2S and the symptomatic transdiagnostic test (S2T), which evaluates 11 clusters of psychiatric symptoms. We assessed the factor structure, internal consistency, invariance, and predictive validity of the revised T2S.

**Results:**

We found a good fit for a bifactor exploratory structural equation modeling (B-ESEM) approach including a global skills factor and seven specific factors. The results also indicate that the new dimension (i.e., control of urges and cravings) has good predictive value, especially for externalizing problems. We also found total invariance of the scale across employment status and partial invariance across the level of psychopathology.

**Conclusion:**

The revised version of the T2S-R has good psychometric properties. It predicts better externalizing problems than the original version. However, the scale remains more correlated with internalizing than externalizing problems. We discuss the implications of the results on the transdiagnostic conceptualization and the interest of using a mixed approach combining transdiagnostic and diagnostic analyses.

## Introduction

Up to now, the diagnostic approach, based on the International Classification of Diseases (ICD; World Health Organization, 2019) and the Diagnostic and Statistical Manual of Mental Disorders (DSM; American Psychiatric Association, 2013), is the main way to conceptualize psychiatric disorders. It is a classification procedure that involves establishing the presence or absence of specific categories with clearly defined properties (Widakowich et al., 2013). This approach has led to the development of gold standard treatments for specific disorders, such as Cognitive Behavior Therapy (CBT) for depression (Haute Autorité de Santé, 2009).

However, several limitations to the diagnostic approach have been observed (Dalgleish et al., 2020). First of all, it has been shown that the essentialist approach cannot be reasonably applied to mental health. For example, Van der Linden (2016) observed that psychiatric disorders do not have specific, inherent properties, as assumed by the diagnostic approach. Moreover, in clinical practice, patients frequently present with non-specific disorders or additional symptoms that cannot be explained directly by their main diagnosis (Zimmerman et al., 2000). The prevalence of comorbidities is also particularly high in psychiatry (Brown et al., 2001). For clinicians seeking to diagnose complex cases, the categorical approach can appear rigid and fixed, failing to take into account the multidimensional nature of psychological mechanisms. The statistical homogenization norms on which current international classifications are based also tend to force clinicians to conceptualize disorders in a particular way (Kammerer and Portelli, 2017). Finally, recent evidence throws into question the reliability of the diagnostic approach. For example, the results of a recent study using artificial intelligence were not consistent with psychiatric nosologies, and the researchers identified only four clusters, with a high level of overlap. All the clusters included six symptoms (i.e., fear, feeling sick, auditory hallucinations, depressed mood, loss of interest, sadness), and each cluster also had specific symptoms (cluster1: sleep and eating problems; cluster 2: repetitive thoughts and actions; cluster 3: feeling isolated and lonely; cluster 4: panic attacks and stress). This is clearly in contradiction with the more than 300 diagnostic categories identified in the DSM-5 (Ghosh et al., 2022).

These observations have led to the development of a new way of conceptualizing psychiatric disorders, namely the transdiagnostic approach. This approach aims to (i) provide a new classification system (e.g., Kotov et al., 2017), and (ii) identify common processes involved in different disorders (e.g., Philippot et al., 2015). The Hierarchical Taxonomy of Psychopathology (HiTOP; Kotov et al., 2017) illustrates the first point, as it proposes a new classification system, based on the view that psychopathology dimensions can be classified at multiple hierarchical levels. According to this view, clinical difficulties are not categorical or binary (present *vs* absent); on the contrary, anybody can have a certain level of difficulty in some of the dimensions, which interact. For example, a patient could present with problems in dimensions such as fear, distress and substance abuse, without meeting all the criteria for multiple anxiety disorders or substance use disorder. The second point, concerning the common processes involved in psychopathology, can be illustrated through Kinderman’s “Mediating Psychological Processes” model (Kinderman, 2005; Kinderman and Tai, 2007). According to Kinderman and Tai (2007), disruption to psychological processes (e.g., rumination) leads to the development of mental disorders. While these processes are considered to be central and proximal mechanisms in psychopathology, mental disorders can also be influenced by biological, social and circumstantial factors, which adversely affect psychological mechanisms. In line with this general model, the Research Domain Criteria (RDoC), developed by the US National Institute of Mental Health (2008), identifies five domains of functioning involved in psychopathology: negative valence systems (e.g., fear), positive valence systems (e.g., reward valuation), cognitive systems (e.g., attention), social systems (e.g., attachment), arousal/regulatory systems (e.g., circadian rhythms). Each set of mechanisms is analyzed at genetic, molecular, cellular, circuits, physiological, behavioral and self-reported levels. A similar conceptualization has also been proposed by Philippot et al. (2015), based on eight psychological mechanisms involved in psychopathology: experiential avoidance, behavioral deactivation, emotion regulation strategies, metacognitive beliefs, low self-efficacy feeling, gaps between the selfs, mental rumination, and intolerance to uncertainty. However, the original theoretical proposal did not test the overlap between these mechanisms, raising many questions for clinical practice, for example the extent to which concepts such as experiential avoidance and emotion regulation strategies overlap.

To fill this gap, a new transdiagnostic tool has recently been developed, the Transdiagnostic Skills Scale (T2S; Vancappel et al., 2022), which aims to evaluate the main psychological skills that are disrupted in psychiatric disorders. This scale enables clinicians to assess the central mechanisms involved in psychopathology with just 42 items, compared to more than 150 items in Philippot et al. (2015) scale. Based on the psychotherapeutic models used in clinical practice, the authors identified six skills that could be developed with patients, involving covert or overt adaptive behaviors: emotion regulation (the ability to reduce the intensity of aversive emotions), behavioral activation and planning (the ability to plan and organize daily activities), emotional identification (the ability to identify and name one’s emotions), assertiveness (the ability to express constructively one’s opinions and needs), problem solving (the ability to solve daily life problems), and emotional confrontation (the ability to face aversive emotions). In the initial study, these skills were negatively related to depression, anxiety, and eating disorders, but not with addictive disorders. A second study (Vancappel et al., 2023) revealed that the skills were negatively related to 10 psychopathological dimensions: negative thoughts and mood, psycho-traumatic and maladaptive symptoms, addictive symptoms, disturbed eating behavior, disturbed perception and behavior, panic and agoraphobia, emotional lability, dejection, neurodevelopmental manifestations, and anxiety. However, while the associations between skills and psychiatric symptoms were high with internalizing symptoms (e.g., anxiety), they were lower with externalizing symptoms (e.g., addictive symptoms). Some authors have suggested that the main mechanisms involved in externalizing disorders is disinhibition (Mullins-Sweatt et al., 2019), defined as an “orientation toward immediate gratification, leading to impulsive behavior driven by current thoughts, feelings, and external stimuli, without regard for past learning or consideration of future consequence” (American Psychiatric Association, 2013, p. 780). Disinhibition would make it difficult for patients to control their cravings or urges, resulting in behavioral problems such as addiction, bulimia, kleptomania or aggressivity. However, the T2S did not include this factor, although it could be determinant in externalizing disorders. This is of prime interest, as previous transdiagnostic programs (e.g., Barlow, 2011) focused mainly on internalizing problems (depression and anxiety) and paid little attention to externalizing problems, limiting their transdiagnostic nature.

The aim of the current study was thus to revise the T2S (T2S-R), adding a new skill, namely the control of cravings and urges (the ability to repress or inhibit cravings and urges), and to assess its factor structure. In this way, the scale is better able to identify a dimension related to externalizing problems. A further aim was to evaluate the invariance of the scale across employment status (students versus workers) and levels of psychopathology (high vs. low) in order to establish whether this scale could be used with different populations. In particular, invariance across employment status would indicate that the T2S-R could be used not only in clinical psychology, but also in fields such as organizational psychology.

## Method

### Participants

We recruited participants online through social media. In order to participate, they had to be at least 18 years of age. Before choosing whether or not to continue with the study, they were asked to read an information note and tick (or sign) a consent form. No compensation was given for participating in the study. The study and consent procedures were approved by the ethics committee of the first author’s university (2022-09-05) and were carried out in accordance with the ethical principles for medical research involving human subjects (World Medical Association, 2013).

### Measures

After providing their informed consent, participants provided socio-demographic information and completed a series of online questionnaires described below.

The *transdiagnostic skills scale 51 items (T2S-51)* is the revised version of the T2S-42 (Vancappel et al., 2022). It is composed of the initial 42 items measuring the first six skills, with nine additional items to assess the control of cravings and urges. These items (e.g., “I can resist my cravings and urges”) were based on questions clinicians typically ask their patients in order to evaluate their ability to control their urges and cravings. The first author proposed the items, which were then reviewed and corrected by clinicians until full agreement was reached. All the initial items were approved by the clinicians, except one, which was reformulated to make it easier to understand. Participants rated each item (initial and new items) on a seven-point Likert scale from 1 (never true) to 7 (always true).

The *symptomatic transdiagnostic test (S2T)* is a self-report questionnaire that measures psychopathology (Vancappel et al., 2023). Sixty-three items measure eleven psychopathological dimensions (i.e., negative thoughts and mood; psycho-traumatic and maladaptive symptoms; addictive symptoms; disturbed eating behavior; disturbed perception and behavior; panic and agoraphobia; emotional lability; dejection; neurodevelopmental manifestations; anxiety; mental hyperactivity). Three additional items measure the functional impairment of the various symptoms. Participants rated each item on a seven-point Likert scale from 1 (never) to 7 (always). Previous studies have found good psychometric properties of the S2T (Vancappel et al., 2023).

### Analyses

Models were estimated using Mplus 8.10 (Muthén and Muthén, 2023) robust maximum likelihood (MLR) estimator. Confirmatory factor analysis (CFA), bifactor-CFA, exploratory structural equation modeling (ESEM), and bifactor-ESEM models (Morin et al., 2016) were performed on participants’ ratings of transdiagnostic skills. In CFA, each item loaded on the factor it was assumed to measure and no cross-loadings were allowed. This model included seven correlated factors representing emotion regulation, behavioral activation and planning, emotional identification, assertiveness, problem solving, emotional confrontation, and control of cravings and urges. In ESEM, the same seven factors were estimated using confirmatory oblique target rotation (Asparouhov and Muthén, 2009). More precisely, all main loadings were specified *a priori* as being freely estimated, while the cross-loadings were constrained to be as close to zero as possible. In bifactor-CFA, all items were allowed to load on one global (G-) factor and seven specific (S-) factors (emotion regulation, behavioral activation and planning, emotional identification, assertiveness, problem solving, emotional confrontation, and control of cravings and urges). No cross-loadings were allowed and all factors were specified as orthogonal according to bifactor assumptions (Chen et al., 2006). In bifactor-ESEM, the same set of G- and S-factors were estimated using orthogonal bifactor target rotation (Reise et al., 2011). More precisely, all items were *a priori* specified as related to the G-factor. In addition, the seven S-factors were defined *a priori* using the same pattern of target and non-target factor loadings used in ESEM.

We assessed model fit (Marsh et al., 2010) using the comparative fit index (CFI), the Tucker-Lewis index (TLI), and the root mean square error of approximation (RMSEA). According to standard interpretation guidelines, values greater than 0.90 and 0.95 for the CFI and TLI, respectively, indicate adequate and excellent fit to the data. Values smaller than 0.08 and 0.06 for the RMSEA indicate, respectively, acceptable and excellent model fit. When comparing nested models, guidelines suggest that models differing from one another by less than 0.01 on the CFI and TLI, or 0.015 on the RMSEA, can be considered to be equivalent (Chen, 2007).

As noted by Morin et al. (2016), fit indices are not sufficient to guide selection of the optimal model. Indeed, unmodelled cross-loadings result in inflated factor correlations in CFA, or inflated G-factor loadings in bifactor-CFA (e.g., Asparouhov et al., 2015). Likewise, an unmodelled G-factor produces inflated factor correlations in CFA, or inflated cross-loadings in ESEM. An examination of parameter estimates is thus required to select the best alternative. As suggested by Morin et al. (2016), model comparison should start by contrasting CFA and ESEM. Here, statistical evidence shows that ESEM provides more exact estimates of factor correlations when cross-loadings are present, while remaining unbiased otherwise (Asparouhov et al., 2015). For this reason, as long as the factors remain well defined, the observation of a distinct pattern of factor correlations supports the ESEM solution. The second step involves contrasting the retained CFA or ESEM solutions with a bifactor alternative. Here, the key elements supporting a bifactor representation are the observation of: (1) an improved level of fit to the data; (2) a well-defined G-factor; and (3) at least some reasonably well-defined S-factors. Observing multiple cross-loadings over 0.100 or 0.200 in ESEM that are lower in bifactor-ESEM is an additional source of evidence in favor of the bifactor solution (Morin et al., 2016).

We also performed correlational analysis to evaluate the concurrent validity of the T2S-R. We considered a correlation above 0.10 as small, above 0.30 as medium, and above 0.50 as strong (Cohen, 1992). For post-traumatic and maladaptive symptoms, we performed the correlations on the whole sample and for the participants who reported a stressful event; participants reporting no such experience had a zero score for this dimension. We assessed the internal consistency of every subscales using Mc Donald’s Omega. Finally, we examined the confidence intervals of the correlations between the different skills and the externalizing difficulties to compare the effect size of the new dimension relative to the other dimensions.

## Results

### Descriptive results

We recruited 1,298 participants (mean age 31.17 ± 13.56), including 1,078 (83.1%) who defined themselves as women, 202 who defined themselves as men (15.6%), and 18 (1.4%) who defined themselves as neither man nor woman. Their education level was as follows: 25 participants (2.0%) had not completed high school, 189 (14.6%) had obtained a high-school diploma, 624 (48.1%) had completed one to 3 years of higher education, 367 (28.3%) had completed four or 5 years of higher education, and 93 (7.2%) had completed more than 5 years of higher education. Concerning their occupational activity, 651 participants (50.2%) were students, 597 (46.0%) were workers, and 110 (8.5%) had another situation (e.g., retired). Details of the descriptive data are displayed in Table 1. We also found a normal distribution of all the continuous variables through scatterplots, allowing the use of parametric tests for inferential analysis.

**Table 1:** Descriptive data.

	Minimum	Maximum	Mean	Standard Deviation	Mc Donald’s ω
T2S-ER	9	62	36.07	9.74	0.92
T2S-BAP	9	63	44.05	8.94	0.89
T2S-EI	6	42	28.95	5.80	0.76
T2S-A	6	42	27.73	5.48	0.72
T2S-PS	6	42	28.48	5.92	0.91
T2S-EC	5	35	21.07	4.89	0.70
T2S-CCU	9	63	42.28	10.16	0.93
T2S-6D	41	276	186.34	32.10	0.95
T2S-7D	50	337	228.62	38.13	0.95
S2T-NTM	5	35	16.64	6.96	0.88
S2T-PMS	7	49	18.10	12.59	0.96
S2T-AS	6	42	14.69	7.99	0.89
S2T-DEB	6	42	17.08	8.67	0.88
S2T-DPB	5	35	9.69	5.02	0.74
S2T-PA	5	35	14.31	7.22	0.87
S2T-EL	7	49	22.95	8.28	0.87
S2T-D	9	49	28.50	7.75	0.85
S2T-NM	4	28	14.19	5.66	0.78
S2T-A	9	63	30.61	11.58	0.88
S2T-PHA	2	14	7.16	2.84	0.41
S2T-FI	3	21	8.02	4.71	0.87
Total S2T	78	462	201.93	63.74	0.96

T2S-ER, emotion regulation; T2S-BAP, Behavioral activation and planning; T2S-EI, emotional identification; T2S-A, assertiveness; T2S-PS, problem solving; T2S-EC, emotional confrontation; T2S-CCU, control of cravings and urges; S2T- NTM negative thoughts and mood; ST2-PMS psycho-traumatic and maladaptive symptoms; ST2-AS, addictive symptoms; S2T-DEB, disturbed eating behavior; S2T-DPB, disturbed perception and behavior S2T-PA, panic and agoraphobia; S2T-EL, emotional lability; S2T-D, dejection; S2T-NM, neurodevelopmental manifestations; S2T-A, anxiety; S2T-PHA, mental hyperactivity; S2T-FI, functional impairment.

The goodness-of-fit of the various measurement models is presented in Table 2. While the CFA showed an acceptable level of fit to the data, the alternative models showed an excellent level of fit across all indicators. In addition, both the ESEM and bifactor-ESEM solutions resulted in substantial increases in model fit when compared to bifactor-CFA (ESEM: ΔCFI = +0.027, ΔTLI = +0.018; bifactor-ESEM: ΔCFI = +0.037, ΔTLI = +0.029). Based on this statistical information, either the ESEM or the bifactor-ESEM solution could be retained. However, as noted above, model selection should be based on a complete examination of parameter estimates and theoretical conformity.

**Table 2:** Goodness-of-fit statistics of the different models.

Description	*χ*^2^ (*df*)	CFI	TLI	RMSEA	90% CI
CFA	3843.148 (1203)*	0.905	0.900	0.041	[0.040, 0.043]
Bifactor-CFA	3108.264 (1173)*	0.931	0.925	0.036	[0.034, 0.037]
ESEM	2106.925 (939)*	0.958	0.943	0.031	[0.029, 0.033]
Bifactor-ESEM	1785.549 (895)*	0.968	0.954	0.028	[0.026, 0.030]

*p < 0.01; CFA, Confirmatory factor analysis; ESEM, Exploratory structural equation modeling; *χ*^2^, Robust chi-square test of exact fit; df, Degrees of freedom; CFI, Comparative fit index; TLI, Tucker-Lewis index; RMSEA, Root mean square error of approximation; 90% CI, 90% confidence interval for the RMSEA.

### Exploratory structural equation modeling versus CFA

The CFA and ESEM models produced well-defined factors with strong loadings. In the ESEM model, many cross-loadings remained either not statistically significant or negligible (only one cross-loading ≥0.300). However, the smaller factor correlations estimated in ESEM (*r* = 0.243 to 0.586) relative to CFA (*r* = 0.315 to 0.718) reinforce the need to incorporate cross-loadings.

### Exploratory structural equation modeling versus bifactor-ESEM

The bifactor-ESEM solution revealed a well-defined G-factor with strong positive loadings for most items (*λ* = 0.316 to 0.765, *ω* = 0.95). In addition, the seven S-factors retained a satisfactory level of specificity: emotional regulation (*λ* = 0.308 to 0.572, *ω* = 0.92), behavioral activation and planning (*λ* = 0.186 to 0.627, *ω* = 0.89), emotional identification (*λ* = 0.321 to 0.625, *ω* = 0.84), assertiveness (*λ* = 0.178 to 0.637, *ω* = 0.72), problem solving (*λ* = 0.329 to 0.378, *ω* = 0.91), emotional confrontation (*λ* = 0.095 to 0.598, *ω* = 0.70), and control of cravings and urges (*λ* = 0.376 to 0.846, *ω* = 0.93). This solution was retained for further analyses. Details of the B-ESEM indexes are displayed in Table 3.

**Table 3:** Standardized factor loadings (*λ*) and uniqueness (*δ*) of the bifactor-ESEM solution.

Items	G *λ*	S-ER *λ*	S-BAP *λ*	S-EI *λ*	S-A *λ*	S-PB *λ*	S-EC *λ*	S-CCU *λ*	*δ*
T2S-ER									
T2S7	**0.568**	**0.524**	0.049	*−0.017*	*−0.039*	*−0.018*	−0.066	*0.022*	0.393
T2S9	**0.570**	**0.572**	0.066	−0.093	*−0.002*	*0.037*	*−0.024*	*0.021*	0.333
T2S11	**0.618**	**0.456**	−0.082	−0.082	*0.036*	0.073	*0.039*	*−0.025*	0.388
T2S12	**0.609**	**0.583**	*0.032*	*−0.004*	*−0.036*	0.051	*−0.010*	*0.020*	0.285
T2S15	**0.540**	**0.337**	*−0.017*	0.075	*0.006*	*0.031*	0.103	0.064	0.574
T2S21	**0.636**	**0.308**	*0.001*	*0.038*	*−0.048*	*−0.060*	*−0.020*	*−0.011*	0.492
T2S33	**0.667**	**0.373**	*−0.032*	*0.022*	*−0.056*	−0.065	*−0.023*	*−0.017*	0.406
T2S36	**0.634**	**0.545**	*0.007*	−0.061	−0.061	*0.027*	*−0.019*	0.052	0.290
T2S42	**0.566**	**0.380**	−0.089	*0.009*	*0.038*	*−0.043*	0.076	*0.019*	0.519
T2S-BAP									
T2S6	**0.530**	0.094	**0.351**	*0.004*	−0.099	*−0.029*	−0.112	*−0.024*	0.563
T2S8	**0.563**	−0.058	**0.563**	−0.078	*−0.008*	−0.074	*−0.038*	*0.033*	0.349
T2S10	**0.545**	0.076	**0.186**	*−0.017*	*0.026*	−0.111	*−0.050*	−0.091	0.639
T2S14	**0.452**	0.064	**0.451**	*0.005*	*0.031*	0.071	*0.036*	0.172	0.551
T2S19	**0.451**	*−0.017*	**0.404**	*−0.030*	*0.050*	0.192	*−0.025*	0.103	0.582
T2S27	**0.568**	−0.089	**0.307**	0.061	*0.035*	0.137	−0.066	*0.048*	0.545
T2S29	**0.495**	*0.005*	**0.600**	*−0.031*	*−0.026*	*0.030*	*0.031*	0.071	0.386
T2S30	**0.566**	*0.051*	**0.345**	*0.003*	*0.005*	*0.027*	*0.019*	*0.030*	0.556
T2S34	**0.588**	−0.052	**0.627**	−0.094	−0.051	*−0.044*	*−0.044*	*0.026*	0.243
T2S-EI									
T2S1	**0.492**	−0.051	−0.087	**0.554**	*−0.010*	*−0.012*	*0.034*	*−0.034*	0.438
T2S2	**0.408**	*0.041*	*0.050*	**0.348**	*−0.060*	−0.076	0.307	*−0.026*	0.603
T2S4	**0.469**	*0.040*	−0.084	**0.321**	*−0.008*	*0.043*	0.100	−0.057	0.653
T2S5	**0.349**	*−0.034*	*0.016*	**0.329**	*0.008*	*0.016*	*0.051*	*−0.025*	0.765
T2S13	**0.610**	*−0.019*	*−0.008*	**0.515**	*0.038*	*0.002*	*0.046*	−0.047	0.356
T2S17	**0.524**	−0.088	−0.087	**0.354**	0.084	*−0.012*	*0.023*	*−0.001*	0.577
T2S31	**0.556**	−0.052	*−0.040*	**0.625**	*−0.036*	*0.030*	*0.011*	*−0.017*	0.293
T2S-A									
T2S16	**0.379**	*−0.038*	−0.066	*−0.019*	**0.474**	*−0.031*	*−0.050*	*0.032*	0.620
T2S23	**0.446**	0.078	0.154	0.089	**0.187**	*0.044*	*0.026*	−0.109	0.714
T2S24	**0.382**	−0.066	−0.057	*−0.043*	**0.637**	*0.006*	*−0.020*	0.062	0.435
T2S25	**0.423**	*−0.017*	−0.049	*−0.005*	**0.565**	*0.000*	*−0.026*	*0.032*	0.498
T2S28	**0.497**	−0.109	*−0.007*	*0.003*	**0.338**	*0.068*	*0.012*	*0.014*	0.622
T2S41	**0.395**	0.076	0.188	0.120	**0.178**	*0.011*	*0.038*	*−0.014*	0.755
T2S-PS									
T2S20	**0.665**	*0.022*	0.079	*0.027*	*0.044*	**0.329**	*0.018*	*−0.013*	0.440
T2S22	**0.650**	0.087	*−0.021*	*0.013*	*0.044*	**0.354**	*−0.028*	−0.051	0.439
T2S32	**0.684**	−0.083	0.061	0.094	*0.035*	**0.330**	−0.055	−0.048	0.397
T2S35	**0.752**	*0.029*	*0.011*	*−0.015*	*−0.005*	**0.371**	−0.047	−0.057	0.290
T2S37	**0.733**	*0.033*	*0.017*	*−0.020*	*−0.010*	**0.377**	*0.000*	*−0.027*	0.318
T2S39	**0.765**	*0.019*	0.081	−0.080	*−0.037*	**0.378**	*0.009*	−0.069	0.252
T2S-EC									
T2S3	**0.377**	−0.065	−0.064	0.164	*0.019*	0.079	**0.310**	*−0.026*	0.720
T2S18	**0.460**	0.194	*0.034*	*0.060*	*0.048*	*0.021*	**0.289**	*0.042*	0.658
T2S26	**0.403**	*0.046*	*−0.008*	*−0.019*	0.202	*0.057*	**0.095**	0.105	0.771
T2S38	**0.358**	−0.103	−0.074	0.124	−0.079	*−0.058*	**0.598**	*−0.012*	0.473
T2S40	**0.480**	*0.042*	−0.055	0.128	*−0.047*	−0.065	**0.572**	*−0.027*	0.415
T2S-CCU									
T2S43	**0.500**	*0.005*	*−0.039*	−0.069	*0.000*	−0.151	*−0.033*	**0.588**	0.375
T2S44	**0.527**	−0.160	−0.198	*−0.016*	*−0.001*	−0.106	−0.104	**0.376**	0.494
T2S45	**0.559**	−0.078	−0.069	−0.075	−0.062	−0.137	*−0.038*	**0.582**	0.308
T2S46	**0.451**	*−0.046*	*−0.024*	*−0.043*	*0.012*	−0.085	−0.062	**0.705**	0.284
T2S47	**0.316**	0.048	0.041	*−0.016*	*0.000*	*−0.020*	*−0.010*	**0.753**	0.328
T2S48	**0.359**	0.067	0.226	*0.025*	*0.036*	0.133	0.094	**0.695**	0.305
T2S49	**0.369**	0.047	0.072	*0.019*	*0.035*	*0.031*	*0.028*	**0.846**	0.138
T2S50	**0.374**	0.134	0.147	*−0.021*	*0.008*	0.070	0.063	**0.708**	0.309
T2S51	**0.385**	*−0.018*	*−0.003*	*0.038*	*0.034*	*0.028*	*−0.016*	**0.669**	0.400

G, Global factor estimated as part of a bifactor model; S, Specific factor estimated as part of a bifactor model; λ, Factor loading; T2S-ER, emotion regulation; T2S-BAP, Behavioral activation and planning; T2S-EI, emotional identification; T2S-A, assertiveness; T2S-PS, problem solving; T2S-EC, emotional confrontation; T2S-CCU, control of cravings and urges; non-significant parameters (*p* ≥ 0.05) are marked in italics.

### Invariance

#### Measurement invariance

The bifactor-ESEM solution was used for tests of measurement invariance. These tests were conducted in the following sequence (Millsap, 2011): (a) configural invariance, (b) weak invariance (loadings), (c) strong invariance (loadings, intercepts), (d) strict invariance (loadings, intercepts, uniqueness), (e) invariance of the latent variance–covariance (loadings, intercepts, uniqueness, variance–covariance), and (f) latent means invariance (loadings, intercepts, uniqueness, variance–covariance, latent means). We tested invariance across employment status (students vs. workers) and across the level of psychopathology (high = S2T > medium vs. low = ST2 ≤ medium). For invariance across employment status, we excluded participants who were both students and workers. The results of these tests are presented in Table 4, and support the measurement invariance of the B-ESEM solution, with the exception of invariance of means across psychopathology levels.

**Table 4:** Goodness-of-fit indices of models for measurement invariance.

Stage	Model and description	SBχ^2^	df	CFI	TLI	RMSEA	RMSEA 90% CI	ΔSBχ^2^(df)	ΔCFI	ΔRMSEA
	Status									
	M1	2828.22	1790	0.959	0.941	0.032	[0.030, 0.034]	–	–	–
	M2	3173.281	2,134	0.959	0.951	0.029	[0.027, 0.031]	375.172 (344)	0	0.003
	M3	3337.028	2,177	0.954	0.946	0.031	[0.029, 0.033]	170.052 (43)*	0.005	−0.002
	M4	4021.658	2,228	0.929	0.919	0.038	[0.036, 0.040]	783.544 (51)*	0.025	−0.007
	M4’	3597.641	2,211	0.945	0.937	0.033	[0.031, 0.035]	254.013 (34)*	0.009	−0.002
	M5	3714.797	2,247	0.942	0.934	0.034	[0.032, 0.036]	89.868 (36)*	0.003	−0.001
	M6	3926.102	2,255	0.934	0.925	0.036	[0.034, 0.038]	275.856 (8)*	0.008	−0.002
	Psychopathological level									
	M1	3038.583	1790	0.950	0.929	0.033	[0.031, 0.035]	–	–	–
	M2	3173.281	2,134	0.953	0.943	0.029	[0.027, 0.031]	357.103 (344)	0.003	−0.004
	M3	3450.377	2,177	0.949	0.941	0.030	[0.028,0.032]	133.865 (43)*	0.004	−0.001
	M4	4251.579	2,228	0.920	0.908	0.037	[0.036, 0.039]	939.562 (51)*	0.029	−0.007
	M4’	3717.385	2,210	0.940	0.931	0.032	[0.031, 0.034]	246.582 (33)*	0.009	−0.002
	M5	3829.983	2,246	0.937	0.929	0.033	[0.031, 0.035]	102.439 (36)*	0.003	−0.001
	M6	4245.600	2,254	0.921	0.911	0.037	[0.035, 0.039]	440.564 (8)*	0.016	−0.004

SB*χ*^2^, Satorra and Bentler (1988) scaled *χ*^2^; df, degrees of freedom; CFI, Comparative fit index; TLI, Tucker-Lewis index; RMSEA, Root mean square error of approximation; RMSEA 90% CI, 90% confidence interval for the RMSEA point estimate; Δ_SB_*χ*^2^(df), change in SB*χ*^2^ and df between models n and n-1; ΔCFI, change in CFI between models n and n-1; ΔRMSEA, change in RMSEA between models n and n-1; all SB*χ*^2^ significant at *p* < 0.001; ^*^ = ΔSB*χ*^2^ significant at *p* < 0.001.

### Correlational analysis

Overall, we found significant moderate to very strong correlations between all the skills and some clusters of symptoms, namely dejection (−0.320 < *r* < −0.688), negative thoughts and mood (−0.326 < *r* < −0.617), anxiety (−0.263 < *r* < −0.596), and emotional lability (−0.330 < *r* < −0.532). We found low/moderate to strong correlations between the skills and other clusters of symptoms, namely post-traumatic and maladaptive symptoms (−0.261 < *r* < −0.485), and panic and agoraphobia (−0.228 < *r* < −0.491). We found low to moderate associations between the skills and some clusters of symptoms, namely disturbed perception and behavior (−0.213 < *r* < −0.434), addictive symptoms (−0.140 < *r* < −0.339), disturbed eating behavior (−0.145 < *r* < −0.325), and neurodevelopmental manifestations (−0.188 < *r* < −0.363). Finally, we found negligible correlations between the skills and mental hyperactivity (−0.08 < *r* < 0.031).

In general, the new dimension of control of cravings and urges had low to moderate/strong correlations (−0.064 < *r* < −0.416) with all the clusters of symptoms, and was the skill that was most correlated with addictive symptoms. The details of the correlation analysis are presented in Table 5.

**Table 5:** Correlations bewteen the T2S and the S2T.

Transdiagnostic Skill	Psychopathological symptom	Correlation	Lower C.I.	Upper C.I.
T2S-A	S2T-NTM	−0.355	−0.402	−0.307
	S2T-PMS (whole sample)	−0.156	−0.208	−0.102
	S2T-PMS (event yes subsample)	−0.233	−0.306	−0.158
	S2T-AS	−0.140	−0.192	−0.086
	S2T-DEB	−0.144	−0.197	−0.090
	S2T-DPB	−0.281	−0.331	−0.230
	S2T-PA	−0.277	−0.327	−0.226
	S2T-EL	−0.330	−0.378	−0.281
	S2T-D	−0.373	−0.419	−0.325
	S2T-NM	−0.343	−0.390	−0.294
	S2T-A	−468	−0.509	−0.424
	S2T-PHA	0.005	−0.049	0.059
	S2T-FI	−0.360	−0.406	−0.312
	Total S2T	−0.390	−0.435	−0.343
T2S-EC	S2T-NTM	−0.326	−0.374	−0.277
	S2T-PMS- (whole sample)	−0.197	−0.249	−0.144
	S2T-PMS (event yes subsample)	−0.316	−0.385	−0.244
	S2T-AS	−0.162	−0.214	−0.108
	S2T-DEB	−0.251	−0.301	−0.199
	S2T-DPB	−0.213	−0.264	−0.160
	S2T-PA	−0.332	−0.380	−0.283
	S2T-EL	−0.330	−0.377	−0.280
	S2T-D	−0.356	−0.403	−0.308
	S2T-NM	−0.216	−0.267	−0.163
	S2T-A	−0.411	−0.456	−0.365
	S2T-PHA	−0.011	−0.065	0.044
	S2T-FI	−0.290	−0.339	−0.239
	Total S2T	−0.385	−0.431	−0.338
T2S-CCU	S2T-NTM	−0.279	−0.328	−0.228
	S2T-PMS (whole sample)	−0.186	−0.238	−0.133
	S2T-PMS (event yes subsample)	−0.261	−0.333	−0.187
	S2T-AS	−0.339	−0.386	−0.290
	S2T-DEB	−0.298	−0.347	−0.247
	S2T-DPB	−0.316	−0.364	−0.266
	S2T-PA	−0.228	−0.279	−0.176
	S2T-EL	−0.416	−0.460	−0.370
	S2T-D	−0.320	−0.368	−0.270
	S2T-NM	−0.188	−0.240	−0.135
	S2T-A	−0.263	−0.313	−0.212
	S2T-PHA	−0.064	−0.118	−0.010
	S2T-FI	−0.289	−0.338	−0.239
	Total S2T	−0.383	−0.428	−0.335
T2S-EI	S2T-NTM	−0.411	−0.455	−0.364
	S2T-PMS (whole sample)	−0.181	−0.233	−0.128
	S2T-PMS (event yes subsample)	−0.304	−0.373	−0.231
	S2T-AS	−0.217	−0.268	−0.164
	S2T-DEB	−0.260	−0.310	−0.209
	S2T-DPB	−0.274	−0.323	−0.222
	S2T-PA	−0.284	−0.333	−0.233
	S2T-EL	−0.330	−0.377	−0.280
	S2T-D	−0.377	−0.422	−0.329
	S2T-NM	−0.231	−0.282	−0.179
	S2T-A	−0.425	−0.469	−0.380
	S2T-PHA	−0.066	−0.120	−0.012
	S2T-FI	−0.290	−0.340	−0.240
	Total S2T	−0.408	−0.452	−0.361
T2S-BAP	S2T-NTM	−0.539	−0.576	−0.499
	S2T-PMS (whole sample)	−0.263	−0.313	−0.211
	S2T-PMS (event yes subsample)	−0.375	−0.440	−0.306
	S2T-AS	−0.341	−0.388	−0.292
	S2T-DEB	−0.259	−0.309	−0.207
	S2T-DPB	−0.434	−0.477	−0.389
	S2T-PA	−0.393	−0.438	−0.346
	S2T-EL	−0.506	−0.545	−0.464
	S2T-D	−0.688	−0.715	−0.658
	S2T-NM	−0.363	−0.409	−0.315
	S2T-A	−0.560	−0.596	−0.521
	S2T-PHA	−0.080	−0.134	−0.026
	S2T-FI	−0.534	−0.572	−0.494
	Total S2T	−0.594	−0.628	−0.557
T2S-ER	S2T-NTM	−0.617	−0.650	−0.582
	S2T-PMS (whole sample)	−0.335	−0.382	−0.286
	S2T-PMS (event yes subsample)	−0.485	−0.543	−0.423
	S2T-AS	−0.273	−0.323	−0.222
	S2T-DEB	−0.325	−0.372	−0.275
	S2T-DPB	−0.358	−0.404	−0.310
	S2T-PA	−0.491	−0.531	−0.448
	S2T-EL	−0.532	−0.570	−0.492
	S2T-D	−0.566	−0.602	−0.528
	S2T-NM	−0.336	−0.384	−0.287
	S2T-A	−0.596	−0.630	−0.560
	S2T-PHA	−0.045	−0.099	0.010
	S2T-FI	−0.441	−0.484	−0.396
	Total S2T	−0.606	−0.640	−0.571
T2S-PS	S2T-NTM	−0.465	−0.506	−0.421
	S2T-PMS (whole sample)	−0.215	−0.266	−0.162
	S2T-PMS (event yes subsample)	−0.357	−0.423	−0.287
	S2T-AS	−0.180	−0.233	−0.127
	S2T-DEB	−0.250	−0.300	−0.198
	S2T-DPB	−0.285	−0.334	−0.234
	S2T-PA	−0.387	−0.432	−0.340
	S2T-EL	−0.401	−0.446	−0.354
	S2T-D	−0.518	−0.557	−0.477
	S2T-NM	−0.264	−0.314	−0.213
	S2T-A	−0.515	−0.554	−0.474
	S2T-PHA	0.031	−0.023	0.085
	S2T-FI	−0.374	−0.420	−0.326
	Total S2T	−0.474	−0.515	−0.431

T2S-ER, emotion regulation; T2S-BAP, Behavioral activation and planning; T2S-EI, emotional identification; T2S-A, assertiveness; T2S-PS, problem solving; T2S-EC, emotional confrontation; T2S-CCU, control of cravings and urges; S2T- NTM negative thoughts and mood; ST2-PMS psycho-traumatic and maladaptive symptoms; ST2-AS, addictive symptoms; S2T-DEB, disturbed eating behavior; S2T-DPB, disturbed perception and behavior S2T-PA, panic and agoraphobia; S2T-EL, emotional lability; S2T-D, dejection; S2T-NM, neurodevelopmental manifestations; S2T-A, anxiety; S2T-PHA, mental hyperactivity; S2T-FI, functional impairment; C.I, Confidence interval.

Finally, we found moderate to strong correlations between the new dimension and the other dimensions of the T2S-R. The details of the results are presented in Table 6.

**Table 6:** Correlations between the different dimensions.

	T2S-ER	T2S-BAP	T2S-EI	T2S-A	T2S-PS	T2S-EC	T2S-CCU
T2S-ER	1						
T2S-BAP	0.570^**^	1					
T2S-EI	0.565^**^	0.439^**^	1				
T2S-A	0.480^**^	0.493^**^	0.454^**^	1			
T2S-PS	0.682^**^	0.661^**^	0.576^**^	0.565^**^	1		
T2S-EC	0.524^**^	0.406^**^	0.577^**^	0.421^**^	0.521^**^	1	
T2S-CCU	0.428^**^	0.445^**^	0.311^**^	0.335^**^	0.393^**^	0.339^**^	1

T2S-ER, emotion regulation; T2S-BAP, Behavioral activation and planning; T2S-EI, emotional identification; T2S-A, assertiveness; T2S-PS, problem solving; T2S-EC, emotional confrontation; T2S-CCU, control of cravings and urges; S2T- NTM negative thoughts and mood; ST2-PMS psycho-traumatic and maladaptive symptoms; ST2-AS, addictive symptoms; S2T-DEB, disturbed eating behavior; S2T-DPB, disturbed perception and behavior; S2T-PA, panic and agoraphobia; S2T-EL, emotional lability; S2T-D, dejection; S2T-NM, neurodevelopmental manifestations; S2T-A, anxiety; S2T-PHA, mental hyperactivity;S2T-FI, functional impairment.

### Comparison of effect size

We assessed the confidence interval of the correlations between the different skills and externalizing symptoms. For addictive symptoms, there was a greater correlation with control of cravings and urges (−0.386 < *r* < −0.290) than with emotional identification (−0.268 < *r* < −0.164), assertiveness (−0.192 < *r* < −0.086), problem solving (−0.233 < *r* < −0.127), and emotional confrontation (−0.214 < *r* < −0.108). For disturbed eating behavior, the only significant difference was a greater correlation with control of cravings and urges (−0.347 < *r* < −0.247) than with assertiveness (−0.197 < *r* < −0.090). For disturbed perception and behavior, the association with control of cravings and urges (−0.364 < *r* < −0.266) was stronger than with emotional confrontation (−0.264 < *r* < −0.160), but weaker than with behavioral activation and planning (−0.477 < *r* < −0.389); the other associations were not significantly different. For emotional lability, the association with control of cravings and urges (−0.460 < *r* < −0.370) was weaker than with emotional regulation (−0.570 < *r* < −0.492); there was no significant difference with the other associations.

## Discussion

The first aim of the study was to evaluate the psychometric properties of the T2S-R, which included the new dimension of control of cravings and urges. Overall, the best fit was a B-ESEM model, composed of one G-factor and seven S-factors. The new dimension fits well in the model. The internal consistency both of this dimension and of the scale as a whole is good. Overall, this suggests that the control of cravings and urges is a transdiagnostic skill. We found that this new dimension significantly predicts the scores of different psychopathologies, with a stronger association for externalizing symptoms and particularly for addictive symptoms. Finally, our findings support a model of latent means invariance across employment status and for a model of latent variance–covariance invariance across levels of psychopathology.

### Future research

The good fit of the B-ESEM model allows scholars and clinicians to analyze both general and specific skills. The invariance across employment status suggests that use of the T2S-R can be extended to other areas such as organizational psychology. However, the invariance is not total across level of psychopathology, and the structure of the scale should be assessed with clinical samples with a higher level of psychopathology. While the addition of the new dimension increases the explanation of externalizing symptoms, overall, the scale has greater predictive power for internalizing problems. This could be due to the greater influence of specific environmental factors on externalizing problems; for example, research has demonstrated the role of drug availability on addiction (Kendler, 2012; Halonen et al., 2013), while other studies have demonstrated the strong impact of sports activities, such as dance, on eating disorders (Ringham et al., 2006; Zoletić and Duraković-Belko, 2009; Herbrich et al., 2011; Francisco et al., 2012). However, to our knowledge, the role of environmental factors on the development of internalizing *vs* externalizing symptoms has not been examined, and further comparative studies should address this gap. Moreover, a model combining the predictive power of skills and environmental factors should be assessed.

The results also support the development of the transdiagnostic approach in psychopathology. We identified one G-factor and seven S-factors, but these factors vary in their influence on cluster symptoms; while each factor was correlated with all the clusters, the main factors differed between clusters. For instance, the control of cravings and urges was the strongest factor for addictive symptoms, while behavioral activation and planning was found to play the strongest role in dejection, and anxiety was best explained by emotional regulation. This suggests variations in the patterns predicting the different disorders. Moreover, the higher level of unexplained variance of externalizing problems in relation to the different skills suggests that specific factors (e.g., environmental factors) are involved in addition to transdiagnostic factors, suggesting that the latter cannot provide a total explanation of psychopathological difficulties. Accordingly, some authors have advocated the development of a bifocal approach combining the analysis of both transdiagnostic and specific/diagnostic factors (Vancappel et al., 2023). The authors suggest that some factors may be transdiagnostic and influence a wide range of psychopathological disorders, while others may be more specific to certain symptoms or clusters of symptoms. For instance, a patient suffering from Post-Traumatic Stress Disorder (PTSD) symptoms and Depersonalization-Derealization Disorder symptoms may present both transdiagnostic and specific factors. For example, emotion regulation may be a transdiagnostic factor that influences both PTSD and dissociative symptoms, while beliefs about dissociation may have a more specific influence on dissociative symptoms. Accordingly, future research should focus on the development of models combining transdiagnostic and diagnostic/specific factors.

### Implications

This study has many implications. At a theoretical level, it seems that transdiagnostic skills can be included in Kinderman and Tai’s model of mental disorder (2007). These skills can be considered as psychological processes that are disturbed in psychopathology. At a clinical level, the results validate the development of a short scale that assesses seven transdiagnostic skills that can be targeted in psychotherapy. This tool avoids the overlap between concepts and the use of multiple scales, which can be time-consuming for patients and clinicians. It also offers a way to assess the skills profile of the patients in order to target the most relevant dimensions. The results suggest that a program targeting the development of these skills may reduce many psychopathological disorders, and an initial trial is currently under way. The invariance of the T2S also suggests that this approach could be used with a wide range of populations, including students and workers, and could thus have applications in fields of psychology other than clinical psychology. The invariance between participants with high *vs* low levels of psychopathology also suggests that the scale can be used with both non-clinical and clinical patients, although further testing of the structure of the scale with a clinical sample is needed. The absence of invariance between latent means when comparing participants with high and low levels of psychopathology is also in line with previous results (Vancappel et al., 2023), as the initial version of the T2S found lower levels of skills in psychiatric patients than in the general population. This is congruent with the idea that these skills are involved in psychopathological difficulties.

### Limitations

This study has some limitations. First, the sample is not completely representative of the general population as it was composed mainly of women and students, hence limiting the generalization of the conclusions. This makes the results particularly open to criticism as there is a higher prevalence of externalizing problems among men than women (Hicks et al., 2007). We also used a non-probabilistic procedure to gather the data, which also limits the representativeness of the general population. Moreover, the use of social media to perform an online study also biases the results, as it excludes participants of a certain age who do not use the Internet. Moreover, administering questionnaires online could also change the way people respond, and future studies should ensure the invariance between pencil-paper and online assessments. Due to the low number of men, we were unable to assess invariance across gender. Future studies are therefore needed with different samples in order to compare the structure and conclusions. Furthermore, the study was cross-sectional in design, so causal relationships could not be established and the results could be explained by common method bias (Podsakoff et al., 2003). Longitudinal or experimental studies should thus be conducted to identify the causal relationships between these psychological skills and psychopathological symptoms.

## Conclusion

The revised version of the T2S (T2S-R) has good psychometric properties. The control of cravings and urges fits the global model well and also has good predictive value for externalizing problems, especially addictive symptoms. This scale is particularly interesting in that it offers a way of measuring a concept involved in multiple psychological disorders. However, overall, the skills are less predictive of externalizing than internalizing problems, suggesting that both transdiagnostic and diagnostic factors are required for a full understanding of psychopathology.

## Data availability statement

The raw data supporting the conclusions of this article will be made available by the authors, without undue reservation.

## Ethics statement

The studies involving humans were approved by Comité d’éthique pour la recherche Tours-Poitiers. The studies were conducted in accordance with the local legislation and institutional requirements. The participants provided their written informed consent to participate in this study.

## Author contributions

AV: Conceptualization, Data curation, Formal analysis, Investigation, Methodology, Project administration, Writing – original draft. NG: Data curation, Formal analysis, Writing – review & editing. EF: Conceptualization, Supervision, Writing – review & editing. SC: Conceptualization, Supervision, Writing – review & editing. JA-B: Conceptualization, Writing – review & editing. HC: Conceptualization, Formal analysis, Writing – review & editing. AM: Conceptualization, Data curation, Writing – review & editing. EJ: Conceptualization, Investigation, Writing – review & editing. CD: Conceptualization, Investigation, Writing – review & editing. WE-H: Conceptualization, Supervision, Writing – review & editing.
